# Proliferative and Invasive Effects of Progesterone-Induced Blocking Factor in Human Glioblastoma Cells

**DOI:** 10.1155/2017/1295087

**Published:** 2017-01-12

**Authors:** Araceli Gutiérrez-Rodríguez, Valeria Hansberg-Pastor, Ignacio Camacho-Arroyo

**Affiliations:** ^1^Unidad de Investigación en Reproducción Humana, Instituto Nacional de Perinatología-Facultad de Química, Universidad Nacional Autónoma de México (UNAM), Ciudad Universitaria, Coyoacán, 04510 Ciudad de México, Mexico; ^2^Facultad de Química, Departamento de Biología, UNAM, Ciudad Universitaria, Coyoacán, 04510 Ciudad de México, Mexico

## Abstract

Progesterone-induced blocking factor (PIBF) is a progesterone (P_4_) regulated protein expressed in different types of high proliferative cells including astrocytomas, the most frequent and aggressive brain tumors. It has been shown that PIBF increases the number of human astrocytoma cells. In this work, we evaluated PIBF regulation by P_4_ and the effects of PIBF on proliferation, migration, and invasion of U87 and U251 cells, both derived from human glioblastomas. PIBF mRNA expression was upregulated by P_4_ (10 nM) from 12 to 24 h. Glioblastoma cells expressed two PIBF isoforms, 90 and 57 kDa. The content of the shorter isoform was increased by P_4_ at 24 h, while progesterone receptor antagonist RU486 (10 *μ*M) blocked this effect. PIBF (100 ng/mL) increased the number of U87 cells on days 4 and 5 of treatment and induced cell proliferation on day 4. Wound-healing assays showed that PIBF increased the migration of U87 (12–48 h) and U251 (24 and 48 h) cells. Transwell invasion assays showed that PIBF augmented the number of invasive cells in both cell lines at 24 h. These data suggest that PIBF promotes proliferation, migration, and invasion of human glioblastoma cells.

## 1. Introduction

Progesterone (P_4_) is a cholesterol-derived steroid hormone that is synthesized by the adrenal glands, gonads, and the central nervous system (CNS) [1]. P_4_ participates in the growth of several types of cancer through the activation of its intracellular receptor (PR), which is a ligand-activated transcription factor [2, 3]. P_4_ promotes the growth, migration, and invasion of human astrocytoma cells lines such as U373 (grade III), U87, and D54 (both grade IV or glioblastomas) [4–6]. In these cell lines, P_4_ effects are mostly mediated by PR, given that the treatment with the receptor antagonist, mifepristone (RU486), almost completely blocks hormone effects. One of the known PR target genes is the progesterone-induced blocking factor (PIBF) that was initially discovered in maternal lymphocytes where it functions as an immunomodulatory factor [7, 8].

Several PIBF isoforms are produced by alternative splicing; the most studied ones are the isoforms of 34, 57, 67, and 90 kDa [9–11]. The latter is the most abundant isoform expressed in most cells, which has been shown to be associated with the centrosome, suggesting a participation in cell cycle regulation [9]. The shorter PIBF isoforms present a tissue-specific expression and have been located in the intra- and extracellular compartments [9, 10]. These short isoforms have been proposed as ligands of the PIBF receptor/interleukin 4 receptor *α* (PIBF-R/IL-4R*α*) heterocomplex that activates diverse proliferative signaling pathways [12, 13]. The most studied pathway is the IL-4R/JAK1/STAT6 [10, 12, 14], associated with the differentiation of Th2 cells, to produce a cytokine specific pattern [15], and with tumor cell growth [16]. Besides, data from our laboratory have demonstrated that PIBF is released to the extracellular compartment and increases the number of U373 cells through the activation of the IL-4R/JAK1/STAT6 pathway [10]. PIBF expression is induced by P_4_ and its production is a common feature during pregnancy [8, 14]. However, it is also synthesized by high proliferative cells such as the trophoblast, mesenchymal stem cells, and tumor cells [9, 14, 17].

PIBF has been associated with cancer due to its proximity to the susceptibility genes of breast cancer in chromosome 13 [9]. Several reports show that PIBF is overexpressed in biopsies of uterus, breast, stomach, and brain cancer [9, 18]. Besides, PIBF is highly expressed in several cancer cell lines derived from astrocytomas (U373) [10], cervix adenocarcinoma (HeLa), chronic myeloid leukemia (K562), ovary adenocarcinoma (OVCAR-3), and breast adenocarcinomas (T47D, SK-BR3, and MCF-7) [9]. In fact, high PIBF concentrations in urine (1000 ng/mL) are observed in patients with malignant tumors, which are almost fourfold higher than those observed during pregnancy (~270 ng/mL) [12, 14]. However, Check et al., 2015, reported that PIBF serum concentrations are not higher in patients with gynecological [19] or breast cancer compared to women with benign tumors [20, 21], possibly due to a mutation of BRCA1 gene that induces the degradation of PR by the proteasome 26S [22]. In these studies, an evaluation of intracytoplasmic PIBF concentrations to elucidate these different findings is suggested.

PIBF expression has been described in astrocytomas [10, 18] which are classified by the World Health Organization (WHO) into four grades (I–IV). Astrocytomas grade IV, also known as glioblastomas, represent the highest evolution grade and also the most frequent malignant brain tumors in humans [23]. Glioblastomas ability to invade several areas of the brain and their short-term recurrence causes the highest rate of death by brain tumors [24]. It has been reported that P_4_ is implicated in the invasive potential of glioblastomas [4]; however, the mechanisms involved in this effect are not completely understood. Interestingly, in a case report, the administration of RU486 resulted in a palliative effect and even extended the patient's life [25]. Glioblastomas are also highly immunosuppressive [26], probably through the secretion of immunomodulatory factors such as TGF-*β* or PIBF [27, 28]. Interestingly, the latter causes a marked diminution of Th1/Th2 cells ratio during pregnancy [15, 29], leading to an immunosuppressive state, which may provide glioma cells with a mechanism of evasion from organism immune system and facilitate tumor progression [30]. Given that PIBF is induced by P_4_ and modulates different pathways involved in cell growth and inflammation, the aim of this study was to investigate the role of PIBF in cell proliferation, migration, and invasion of U87 and U251 cells derived from human glioblastomas.

## 2. Materials and Methods

### 2.1. Cell Culture

U87 and U251 (ATCC, VA, USA) cells derived from human glioblastomas were cultured in Dulbecco's modified eagle medium (DMEM) with phenol red, supplemented with fetal bovine serum (FBS) (10%), pyruvate (1 mM), glutamine (2 mM), and nonessential amino acids (0.1 nM) (Biowest, Nuaillé, FRA); the culture was maintained at 37°C, under 95% humidity/5% CO_2_ atmosphere. Cells were grown until reaching a 70–80% confluence.

### 2.2. Treatments

U87 and U251 cells were grown in phenol red-free DMEM medium (In Vitro S.A., CDMX, MEX) supplemented with FBS (10%) without hormones 24 hours before the following treatments: vehicle (cyclodextrin 0.02%), P_4_ coupled to cyclodextrin (10 nM), PR antagonist RU486 (10 *μ*M), and the combined treatment of P_4_ plus RU486. Recombinant PIBF (100 and 200 ng/mL) (Abnova, TP, TWN) was used for treating cells in the cell counting, proliferation, wound-healing, and transwell assays. Phenol red and hormone-free DMEM was used as the vehicle in these experiments. Recombinant PIBF shows isoforms with approximate molecular weights of 95, 60, and 40 kDa according to the manufacturer's specifications.

### 2.3. Total RNA Extraction and RT-PCR

To determine the effect of P_4_ on PIBF gene expression, 5 × 10^5^ cells were grown as described in the “Cell Culture” section and treated for 6, 12, and 24 h. Total RNA was isolated from U87 cells by the method based on guanidine isothiocyanate/phenol/chloroform extraction according to the TRIzol reagent manufacturer's protocol (Invitrogen, CA, USA). Isolated RNA was quantified using the spectrophotometer Nanodrop-2000 (Thermo Scientific, MA, USA) at 260 nm. 1 *μ*g of total RNA was used to synthesize the first-strand cDNA with the M-MLV reverse transcriptase (Thermo Scientific, MA, USA) following the manufacturer's instructions. 2 *μ*L of synthesized cDNA was used to amplify PIBF and the internal control 18S ribosomal RNA (rRNA) with the following specific primers: 5′-GACAGAGCCAATTCGCTATTAAACCAGACTCAACAGC-3′ in the sense primer and 5′-GCTGAGTACACGATTAAGCTGAATTTTGTTTTCCATCAG-3′ in the antisense for PIBF, and for 18S amplification the sequences were 5′-CGCGGTTCTATTTTGTTGGT-3′ in the sense and 5′-AGTCGGCATCGTTTATGGTC-3′ in the antisense (Sigma-Aldrich, MO, USA). Negative controls without cDNA and with nonretrotranscribed RNA were included in all experiments. The PCR reaction was performed as follows: an initial PCR activation step at 94°C (5 min), 25 cycles of denaturation at 94°C (30 s), annealing at 68°C (30 s), and elongation at 72°C (30 s). A final extension cycle was performed at 72°C (5 min). The PCR products were separated by electrophoresis on a 1.5% agarose gel at 70 V for 120 min and stained with GelRed™ (Biotium, CA, USA). The gel image was captured under a UV transilluminator and analyzed for band densitometry using the ImageJ software (National Institute of Health, WA, USA). PIBF expression level was normalized to that of the internal control 18S rRNA.

### 2.4. Western Blotting

To determine the effect of P_4_ on PIBF protein content in the U87 cell line, 5 × 10^5^ cells were seeded in 6-well plates and treated for 12 and 24 h as described in the “Treatments” section. Total proteins were extracted with RIPA lysis buffer (150 nM NaCl, 50 nM Tris-HCl, 1 mM EDTA, 1% Triton, and 0.1% SDS) and quantified using the spectrophotometer Nanodrop-2000 (Thermo Scientific, MA, USA) at 280 nm. 70 *μ*g of total lysate protein was separated on 7.5% SDS-PAGE at 70 V for 2 h. The proteins were transferred to nitrocellulose membranes (Millipore, MA, USA) at 60 mA for 2 h in semidry conditions. After blocking with 3% fat-free milk and 2% bovine serum albumin (BSA) at 4°C overnight, the membrane was incubated with an antibody against PIBF (1 *μ*g/mL, sc-99129, Santa Cruz Biotechnology, TX, USA) at 4°C overnight. Blots were then incubated with an anti-rabbit secondary antibody conjugated to horseradish peroxidase (0.2 *μ*g/mL, sc-2004, Santa Cruz Biotechnology, TX, USA) for 45 min at room temperature. In order to correct the differences in the amount of total protein loaded, membranes were incubated with glycine (0.1 M, pH 2.5, 0.5% SDS) for 45 min at room temperature and afterwards with the *α*-tubulin primary antibody (0.5 *μ*g/mL, sc-5286, Santa Cruz Biotechnology, TX, USA) at 4°C overnight. Blots were incubated with an anti-mouse secondary antibody conjugated to horseradish peroxidase (0.2 *μ*g/mL, sc-2005, Santa Cruz Biotechnology, TX, USA) for 45 min. Chemiluminescence signals were detected exposing the membranes to Kodak Biomax Light Films (Sigma-Aldrich, MO, USA) using the peroxidase substrate SuperSignal West Femto Maximum Sensitivity (Thermo Scientific, MA, USA). Blot images were captured using a Canon digital camera and the bands intensities were quantified with the ImageJ software (National Institute of Health, WA, USA).

### 2.5. siRNA Transfection

To determine the specificity of PIBF bands obtained by Western Blot, PIBF silencing was performed as follows: 1 × 10^5^ U87 cells were seeded in 6 wells in DMEM without antibiotics, and 24 h later the medium was replaced with phenol red and antibiotics-free DMEM, with a control siRNA that has no specific mRNA target sequence, and a PIBF siRNA (75 nM) (Santa Cruz Biotechnology, TX, USA), using the transfection reagent Lipofectamine RNAiMAX (Thermo Scientific, MA, USA). Cells were harvested 48 h after the transfection and a Western Blot analysis was performed as described in the “Western Blotting” section.

### 2.6. Cell Counting

1 × 10^4^ U87 cells were seeded in 24-well plates and grown for 24 h; cells were treated for 5 days as described in “Treatments” section and harvested from incubation each day. The trypan blue dye exclusion assay was used to test the effect of PIBF on cell growth and viability, using a Neubauer chamber and an inverted microscope CKX41 (Olympus, TYO, JPN) at 40x magnification.

### 2.7. Cell Proliferation Assay

5-bromo-2′-deoxy-uridine (BrdU) incorporation assay was used to test the proliferative effect of PIBF. 4 × 10^3^ U87 cells were seeded in chambered cell culture slides and maintained as described in the “Cell Culture” section. After 24 h, the medium was replaced for phenol red and hormone-free DMEM medium. Cells were treated for 4 days as described in “Treatments” section. After treatments, the bromo-2′-deoxy-uridine Labeling and Detection Kit I (Roche, BW, DE) was used according to the manufacturer's protocol. Hoechst 33342 Fluorescent Dye (Thermo Scientific, MA, USA) was used to stain the DNA. The fluorescence signal was observed at dual-wave lengths of 486 and 515–565 nm in the fluorescence microscope Olympus Bx43F (Olympus, TYO, JPN). The number of cells with BrdU incorporation was obtained using the ImageJ software (National Institute of Health, WA, USA) and the percentage of cells positive for BrdU was calculated considering the total number of cells stained with Hoechst.

### 2.8. Migration Assay

The wound-healing assay was used to examine the effect of PIBF on glioblastoma cell migration. 3 × 10^5^ U87 cells and 2 × 10^5^ U251 cells were seeded in six-well plates and grown until reaching a 70–80% confluence; then the medium was changed for phenol red-free DMEM medium supplemented with fetal bovine serum (10%) without hormones. 24 h later, when the monolayer was absolutely confluent, two parallel scratches by well were made with a 200 *μ*L pipet tip. The detached cells were washed by aspiration. Cells were incubated with DNA synthesis inhibitor cytosine *β*-D-arabinofuranoside hydrochloride (10 *μ*M, Ara-C; Sigma-Aldrich, MO, USA) for 1 h prior to PIBF treatments to avoid cell proliferation. Images of the scratch area were captured with an Infinity 1-2C camera (Lumenera, ON, CAN) attached to an inverted microscope CKX41 (Olympus, TYO, JPN) at 40x magnification at 0, 3, 6, 12, 24, and 48 h. At 24 h medium, treatments, and Ara-C were replaced. Three random fields were selected to count the number of cells that migrated in the scratch area using the ImageJ software (National Institute of Health, WA, USA).

### 2.9. Invasion Assay

To evaluate the effect of PIBF on cell invasion, transwell invasion assays were performed. 1 mL of Matrigel (Sigma-Aldrich, MO, USA), diluted in phenol red and FBS-free DMEM at a final concentration of 2 mg/mL, was added to the transwell inserts (8.0 *μ*m membrane; Corning, USA) placed on six-well plates and incubated for 24 hours at 37°C, under 95% humidity/5% CO_2_ atmosphere. 4 × 10^5^ U87 cells and 3 × 10^5^ U251 cells cultured in phenol red and hormone-free DMEM medium were suspended in 1.5 mL of phenol red and FBS-free DMEM that included 100 ng/mL of recombinant PIBF and 10 *μ*M Ara-C. Cells were plated on the matrigel and phenol red-free DMEM medium supplemented with fetal bovine serum (10%) with hormones was added to the bottom of the well as chemoattractant. The plate was incubated for 24 h, then the matrigel was removed, and the cells were fixed with paraformaldehyde (4%) and stained with crystal violet (1%). Images of 5 random fields of each insert were captured with an Infinity 1-2C camera (Lumenera, ON, CAN) attached to an inverted microscope CKX41 (Olympus, TYO, JPN) at 40x magnification, and the number of invasive cells was counted using the ImageJ software (National Institute of Health, WA, USA).

### 2.10. Statistical Analysis

All data were analyzed and plotted using the GraphPad Prism 5.0 software (GraphPad Software, CA, USA). A one-way ANOVA was used for Western Blot experiments and a two-way ANOVA was used for cell counting and migration assays, followed by the Bonferroni post hoc test in all cases. Data from the RT-PCR, proliferation, and invasion assays were analyzed by a two-tail unpaired Student's *t*-test. A value of *p* < 0.05 was considered statistically significant as stated in the figure legends.

## 3. Results

### 3.1. PIBF Gene Expression Is Regulated by P_4_

**Figure 1:**
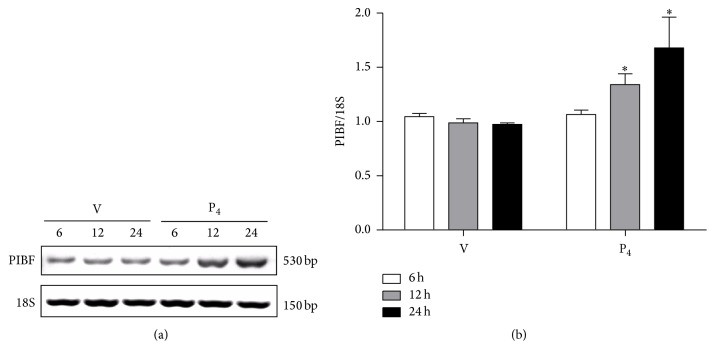
P_4_ regulates PIBF expression in glioblastoma cells. PIBF gene expression was evaluated by RT-PCR in U87 cells treated with vehicle (V, cyclodextrin 0.02%) and P_4_ (10 nM) for 6, 12, and 24 h. (a) Representative image of PIBF gene expression (530 bp) at different times of treatment and the respective expression control gene 18S rRNA (150 bp). (b) Densitometric analysis of three independent experiments; PIBF expression values were normalized to those of the control gene 18S rRNA. The data are expressed as the mean ± S.E.M. with *n* = 3; ^*∗*^*p* < 0.05 versus vehicle.

To assess the P_4_-mediated regulation of PIBF in U87 human glioblastoma cell line, we performed RT-PCR using cells treated with vehicle (cyclodextrin 0.02%) and P_4_ (10 nM) for 6, 12, and 24 h. A specific fragment of 530 bp was amplified in U87 cells. P_4_ treatment did not modify PIBF expression at 6 h, but at 12 and 24 h it significantly increased its expression when compared to the vehicle (Figures 1(a) and 1(b)).

### 3.2. P_4_ Upregulates PIBF 57 kDa Isoform Content

**Figure 2:**
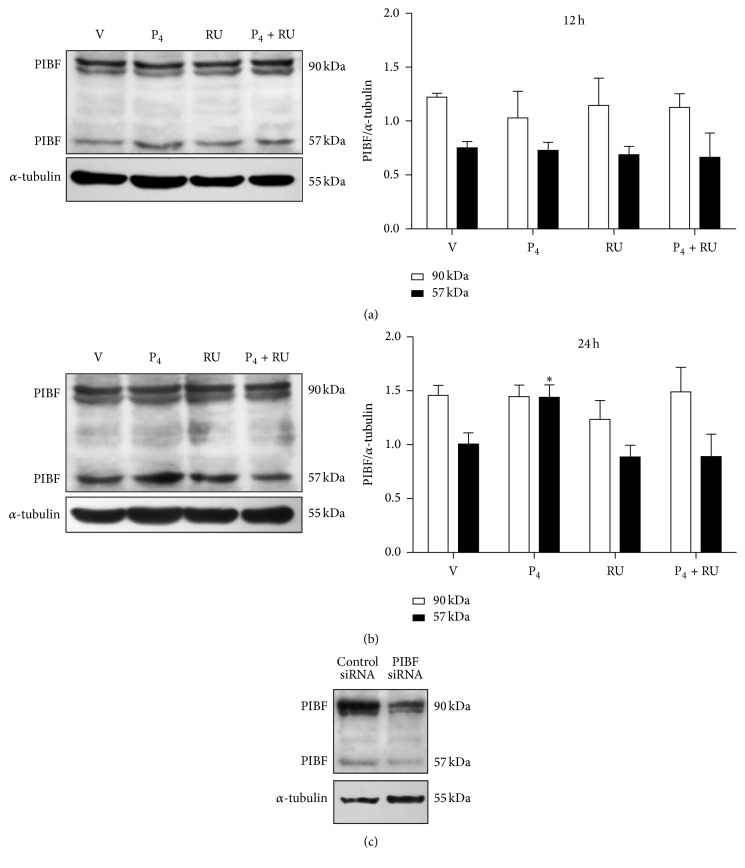
PIBF (57 kDa) isoform is regulated by P_4_ in glioblastoma cells. Western Blot for PIBF protein was performed in U87 cells treated with vehicle (V, cyclodextrin 0.02%), P_4_ (10 nM), RU486 (10 *μ*M), and P_4_ plus RU486 (P_4_ + RU) for 12 and 24 h. Representative images of PIBF isoforms content are shown with their respective densitometric analysis after 12 h (a) and 24 h (b) of treatment. For the densitometric analysis, PIBF values were corrected with those of the internal control, *α*-tubulin. The data are expressed as the mean ± S.E.M. with *n* = 4; ^*∗*^*p* < 0.05 versus the other treatment groups. (c) PIBF expression was silenced using a specific siRNA and a control siRNA that lacks any known mRNA target sequence. The image shows the reduction of both PIBF isoforms as evaluated by Western Blot.

Several PIBF isoforms are produced by alternative mRNA splicing and particularly the 90 kDa isoform is frequently overexpressed in cancer cells [9, 10, 18]. We first evaluated if PIBF isoforms were expressed in U87 cells and also if their content was regulated by P_4_. We detected by Western Blot two main isoforms, the largest one of 90 kDa and a shorter one of 57 kDa (Figure 2). In U87 cells, the 90 kDa isoform was the most abundant one. P_4_ treatment had no effects on PIBF isoforms content at 12 h (Figure 2(a)), while at 24 h we observed an increase in the content of the 57 kDa isoform that was blocked by RU486 (Figure 2(b)), suggesting its regulation through PR. The silencing of both PIBF isoforms with siRNAs reduced the intensity of PIBF bands 70%, corroborating the specificity of the bands recognized by the used antibody (Figure 2(c)).

### 3.3. Cell Number and Proliferation Are Increased by PIBF

**Figure 3:**
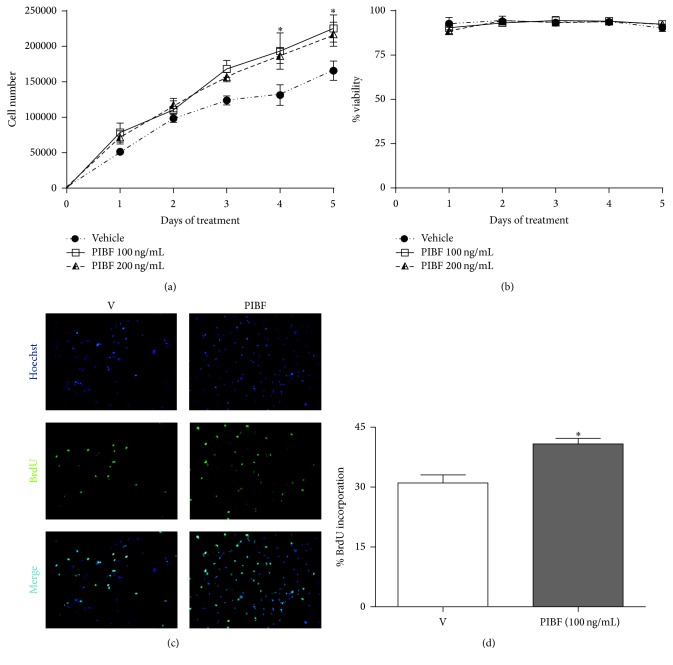
PIBF increases proliferation and the number of U87 cells. Cells were treated with recombinant PIBF (100 and 200 ng/mL) during 5 days, while the medium was used as the vehicle (V). Cells were harvested each day to determine the number of cells (a) and the cell viability (b) by using the trypan blue dye exclusion method. Cell proliferation was measured after the treatment of PIBF (100 ng/mL) during 4 days. Representative immunofluorescence images of cell nuclei (Hoechst stain, upper panel), BrdU positive cells (middle panel), and the merged image (lower panel) are shown (c). The graph shows the percentage of cells incorporating BrdU (d). The data were obtained from three independent experiments and are expressed as the mean ± S.E.M. ^*∗*^*p* < 0.05 versus vehicle.

PIBF (100 and 200 ng/mL) increased the number of cells on days 4 (47 and 42%, resp.) and 5 (36 and 31%, resp.) (Figure 3(a)) as compared with vehicle. None of the treatments had any effect on cell viability (Figure 3(b)). A BrdU incorporation assay was utilized in order to investigate whether the increase in the number of U87 cells by PIBF was due to an augment in cell proliferation. The low PIBF concentration (100 ng/mL) was used in these experiments and administered during 4 days. PIBF treatment enhanced by 30% the BrdU incorporation in U87 cells (Figures 3(c) and 3(d)).

### 3.4. PIBF Induces Cell Migration

**Figure 4:**
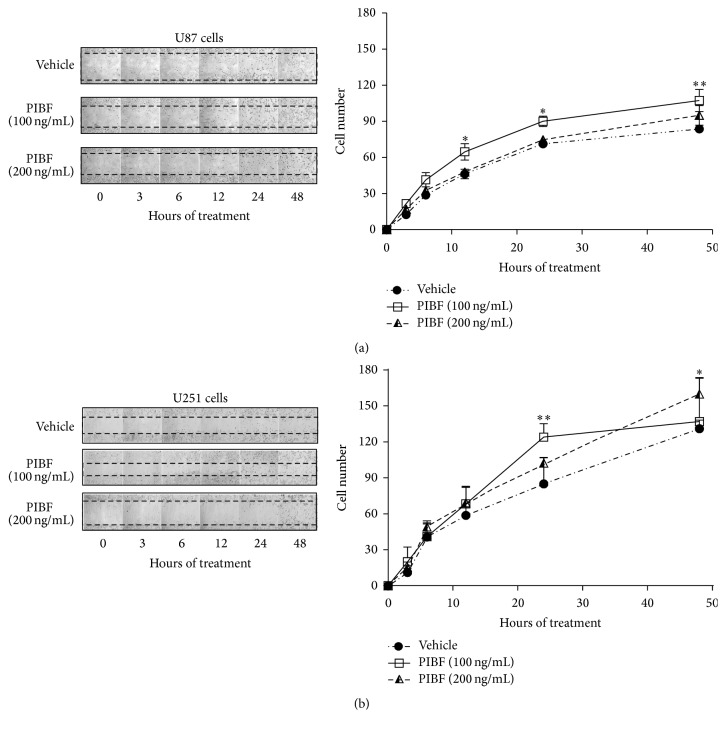
Migration of glioblastoma cells is enhanced by PIBF. Wound-healing assay was performed to evaluate the migration capabilities of U87 and U251 cells upon PIBF exposure. Cells were treated with the recombinant PIBF (100 and 200 ng/mL) for 48 h while the medium was used as the vehicle (V). (a) and (b) show representative images of the wound areas taken at 0, 3, 6, 12, 24, and 48 h after treatments in U87 and U251 cells, respectively, in the left panels. The graphs represent the number of cells that migrated into the wound area at the given time of treatment; the data were obtained from four independent experiments and are expressed as the mean ± S.E.M.; ^*∗*^*p* < 0.01 and ^*∗∗*^*p* < 0.001 versus vehicle.

To evaluate if PIBF modifies the migration capabilities of U87 and U251 cells, a wound-healing assay was performed. During the experiment, the DNA synthesis inhibitor Ara-C was used to discard that an increase in the number of migrating cells was due to cell proliferation. The treatments of PIBF (100 and 200 ng/mL) were added for 48 h, and the respective images of the migrating cells were taken at 0, 3, 6, 12, 24, and 48 h (Figures 4(a) and 4(b)). PIBF (100 ng/mL) increased the number of U87 and U251 migrating cells from 12 to 48 h and at 24 h, respectively. Interestingly, the highest concentration of PIBF (200 ng/mL) augmented the number of migrating U251 cells at 48 h, without effects on U87 cells (Figures 4(a) and 4(b)).

### 3.5. PIBF Increases the Number of Invasive Cells

**Figure 5:**
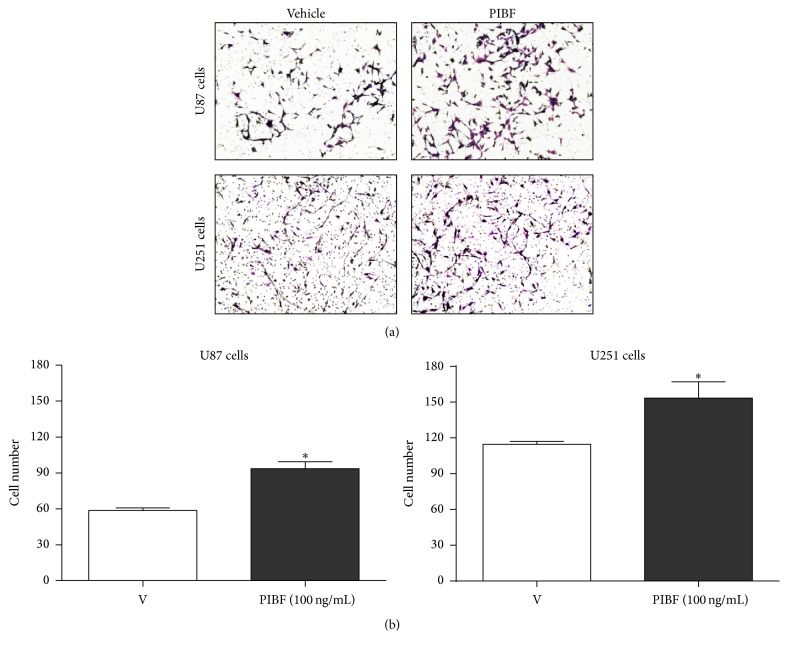
PIBF induces invasion of U87 and U251 cells. The number of invasive cells after PIBF treatment was evaluated by a transwell invasion assay. Cells were treated with recombinant PIBF (100 ng/mL) for 24 h, while the medium was used as the vehicle. (a) Representative images of the invasive cells after PIBF treatment in both cell lines. (b) The graph shows the number of invasive cells from four independent experiments. The data are expressed as the mean ± S.E.M.; ^*∗*^*p* < 0.01 versus vehicle.

To evaluate the effect of PIBF on the invasion capability of U87 and U251 cells, we used the concentration of 100 ng/mL that induced an increase in cell migration in both cell lines at 24 h. Transwell assays were performed with cells treated with PIBF for 24 h; the medium was used as the vehicle. The images were captured from five random fields as it is shown in Figure 5(a). As in the case of migration, PIBF significantly increased the number of U87 (50%) and U251 (30%) invasive cells, in comparison with the vehicle (Figures 5(a) and 5(b)).

## 4. Discussion

Glioblastoma is the most common and aggressive brain tumor in humans; it has a high rate of mortality due to its uncontrollable growth, its ability to infiltrate the adjacent tissue, and its short-term recurrence [24]. The current treatments neither improve the patient's life quality nor extend their survival time [31, 32]. Data from our laboratory have demonstrated that P_4_ induces both in vitro and in vivo glioblastoma cell proliferation and invasion partly through the activation of PR [4–6], but the mechanisms implicated in these effects are not well understood. There is evidence that very high concentrations of P_4_ (40 and 80 *μ*M) alone or in combination with temozolomide (TMZ, 50 and 100 *μ*M), the most used chemotherapeutic agent for glioma treatment, decrease the proliferation and migration in different cell lines derived from human glioblastomas, and this decrease was not blocked by RU486. However, in the same studies, lower concentrations (0.1 to 5 *μ*M) of P_4_ increase cell viability/proliferation of human glioblastoma cells through PR activation [33, 34], consistent with our previous reports. These differential effects could be due to the activation of distinct signaling mechanisms by P_4_, depending on its concentration. Further experiments with low and high concentrations of P_4_ and PIBF, alone and with TMZ, are needed to elucidate this opposed effect.

There are reports that show an increase of PIBF serum concentration induced by P_4_ in both healthy women and those with different gynecological diseases [35]. In contrast, there is evidence indicating that, in MCF-7 breast cancer cells, P_4_ is not able to increase the expression of PIBF [9]. In this study, we demonstrated that P_4_ significantly increases PIBF gene expression at 12 h and this effect lasted for 24 h. Interestingly, P_4_ increases the expression of PIBF from 1 h to 24 h in U373 human astrocitoma cell line (now known as U251 glioblastoma cell line after a short tandem repeat-PCR genotyping) [10], indicating a differential hormone regulation of PIBF expression depending on cell context in human glioblastomas.

PIBF has several isoforms produced by alternative splicing [9, 36] but in tumor cells; these isoforms have not been fully characterized. In U87 cells we observed two isoforms: the 90 kDa protein encoded by the full-length mRNA and a shorter isoform of 57 kDa, both found by González-Arenas et al. (2014) in astrocytoma cells as well [10]. The largest isoform is mainly localized in the nucleus, and it is associated with the centrosome, while the other shorter isoforms have been found in the intra- and extracellular compartments, and it is suggested they function in an autocrine mode [9, 10]. In U87 cells we did not observe changes in the content of the full-length PIBF protein upon treatment with P_4_, but it increased the 90 kDa protein content in U373 cells (now known as U251) [10]. Remarkably, the content of 57 kDa protein was upregulated at 24 h by P_4_ in U87 cells but not in U251 cells [10]. In both tumor cell lines the inducing effect of the hormone was blocked by the PR antagonist RU486, suggesting its regulation through PR. Further experiments are needed to evaluate the processes involved in this differential regulation of PIBF expression depending on the glioblastoma cell line.

PIBF expression has been described in several cells with a high proliferation rate [9, 17, 37]. It has been suggested that PIBF isoforms act as ligands implicated in the activation of proliferation signaling pathways including JAK1/STAT6 [9, 10, 12, 38], through the interaction with a receptor heterocomplex consisting of PIBF-R, which is attached to the plasma membrane by a glycosylphosphatidylinositol [13], and membrane receptor IL-4R*α* [10, 12]. In previous studies, PIBF (200 ng/mL) increased the number of astrocytoma cells through the activation of the IL-4R*α*/JAK1/STAT6 pathway [10]. In this work, we observed that PIBF induced cell proliferation, which was reflected in an increase in the number of U87 cells, probably by activating this same pathway.

The activation of JAK2/STAT3 pathway, which is overactivated in glioblastomas, promotes cell migration and invasion in several types of cancer [39, 40]. Interestingly, in the HT-1080 human fibrosarcoma cell line, it has been reported that PIBF is implicated in the augment of STAT3 phosphorylation [11]. In this study, we observed that PIBF (100 ng/mL) enhances the migration of glioblastoma cells at 12 and 24 h in U87 and U251 cells, respectively. Interestingly, chromatin immunoprecipitation assays in HT-1080 cells demonstrated that PIBF binds the promoter regions of IL-6 [11], an overexpressed cytokine in glioblastomas that acts as a ligand of the JAK2/STAT3 pathway [26, 41]. Therefore, the overactivation of this pathway by PIBF could be a possible mechanism underlying its effects on cell migration.

PIBF also binds the promoter region of epidermal growth factor (EGF) gene in human fibrosarcoma cells [11]. EGF through its receptor (EGFR), which is frequently overexpressed in glioblastomas [42], promotes the transcription of genes associated with the extracellular matrix proteolysis such as matrix metalloproteases 2 and 9 (MMP-2 and 9) [11]. In this study, we observed that the treatment of PIBF (100 ng/mL) augmented the invasive capability of both cell lines at 24 h. Taking into consideration that PIBF acts as a transcriptional factor [11], we suggest a possible role of PIBF in regulating the expression of several target genes such as IL-6 and EGF that could be associated with the invasive effects of glioblastoma cells. However, it is still unknown which of the PIBF isoforms promote the cell migration and invasion of glioblastoma cells.

## 5. Conclusions

In summary, our data suggest that P_4_ differentially upregulates PIBF expression by PR activation depending on the glioblastoma cell line and that PIBF increases glioblastoma cell proliferation, migration, and invasion, indicating an important role of this protein in the regulation of the mechanisms involved in tumor progression.

## References

[1] Schumacher M., Guennoun R., Robert F. (2004). Local synthesis and dual actions of progesterone in the nervous system: neuroprotection and myelination. *Growth Hormone and IGF Research*.

[2] Rodriguez-Dorantes M., Camacho-Arroyo I. (2006). Transcriptional activity regulated by progesterone receptor isoforms. *Molecular Endocrinology*.

[3] Cabrera-Muñoz E., González-Arenas A., Saqui-Salces M. (2009). Regulation of progesterone receptor isoforms content in human astrocytoma cell lines. *Journal of Steroid Biochemistry and Molecular Biology*.

[4] Piña-Medina A. G., Hansberg-Pastor V., González-Arenas A., Cerbón M., Camacho-Arroyo I. (2016). Progesterone promotes cell migration, invasion and cofilin activation in human astrocytoma cells. *Steroids*.

[5] Germán-Castelán L., Manjarrez-Marmolejo J., González-Arenas A., González-Morán M. G., Camacho-Arroyo I. (2014). Progesterone induces the growth and infiltration of human astrocytoma cells implanted in the cerebral cortex of the rat. *BioMed Research International*.

[6] González-Agüero G., Gutiérrez A. A., González-Espinosa D. (2007). Progesterone effects on cell growth of U373 and D54 human astrocytoma cell lines. *Endocrine*.

[7] Szekeres-Bartho J., Autran B., Debre P., Andreu G., Denver L., Chaouat G. (1989). Immunoregulatory effects of a suppressor factor from healthy pregnant women's lymphocytes after progesterone induction. *Cellular Immunology*.

[8] Druckmann R., Druckmann M.-A. (2005). Progesterone and the immunology of pregnancy. *Journal of Steroid Biochemistry and Molecular Biology*.

[9] Lachmann M., Gelbmann D., Kálmán E. (2004). PIBF (progesterone induced blocking factor) is overexpressed in highly proliferating cells and associated with the centrosome. *International Journal of Cancer*.

[10] González-Arenas A., Valadez-Cosmes P., Jiménez-Arellano C., López-Sánchez M., Camacho-Arroyo I. (2014). Progesterone-induced blocking factor is hormonally regulated in human astrocytoma cells, and increases their growth through the IL-4R/JAK1/STAT6 pathway. *Journal of Steroid Biochemistry and Molecular Biology*.

[11] Halasz M., Polgar B., Berta G., Czimbalek L., Szekeres-Bartho J. (2013). Progesterone-induced blocking factor differentially regulates trophoblast and tumor invasion by altering matrix metalloproteinase activity. *Cellular and Molecular Life Sciences*.

[12] Kozma N., Halasz M., Polgar B. (2006). Progesterone-induced blocking factor activates STAT6 via binding to a novel IL-4 receptor. *The Journal of Immunology*.

[13] de la Haba C., Palacio J. R., Palkovics T., Szekeres-Barthó J., Morros A., Martínez P. (2014). Oxidative stress effect on progesterone-induced blocking factor (PIBF) binding to PIBF-receptor in lymphocytes. *Biochimica et Biophysica Acta (BBA)—Biomembranes*.

[14] Szekeres-Bartho J., Polgar B. (2010). PIBF: the double edged sword. Pregnancy and tumor. *American Journal of Reproductive Immunology*.

[15] Szekeres-Bartho J., Halasz M., Palkovics T. (2009). Progesterone in pregnancy; receptor-ligand interaction and signaling pathways. *Journal of Reproductive Immunology*.

[16] Merk B. C., Owens J. L., Lopes M.-B. S., Silva C. M., Hussaini I. M. (2011). STAT6 expression in glioblastoma promotes invasive growth. *BMC Cancer*.

[17] Anderle C., Hammer A., Polgár B. (2008). Human trophoblast cells express the immunomodulator progesterone-induced blocking factor. *Journal of Reproductive Immunology*.

[18] Kyurkchiev D., Naydenov E., Tumangelova-Yuzeir K. (2014). Cells isolated from human glioblastoma multiforme express progesterone-induced blocking factor (PIBF). *Cellular and Molecular Neurobiology*.

[19] Check J. H., Sarumi M., DiAntonio A., Hunter K., Simpkins G., Duroseau M. (2015). Serum levels of the progesterone induced blocking factor do not precipitously rise in women with gynecologic cancer in contrast to women exposed to progesterone. *Clinical and Experimental Obstetrics and Gynecology*.

[20] Check J. H., Rosenberg A., DiAntonio A., Rui H., Cohen R., DiAntonio G. (2015). Abstract 1281: serum levels of the immunomodulatory protein, the progesterone induced blocking factor (PIBF) are not higher in women with progesterone (P) receptor (R) positive vs. negative breast cancer. *Cancer Research*.

[21] Check J. H., Dougherty M. P., DiAntonio G., Vaniver J., Duroseau M., Srivastava M. D. (2015). Abstract 1282: comparison of serum progesterone levels of the immunomodulatory protein, the progesterone induced blocking factor, in people with BRCA-2 mutations associated with and not associated with a high risk of cancer. *Cancer Research*.

[22] Calvo V., Beato M. (2011). BRCA1 counteracts progesterone action by ubiquitination leading to progesterone receptor degradation and epigenetic silencing of target promoters. *Cancer Research*.

[23] Louis D. N., Perry A., Reifenberger G. (2016). The 2016 world health organization classification of tumors of the central nervous system: a summary. *Acta Neuropathologica*.

[24] Furnari F. B., Fenton T., Bachoo R. M. (2007). Malignant astrocytic glioma: genetics, biology, and paths to treatment. *Genes and Development*.

[25] Check J. H., Wilson C., Cohen R., Sarumi M. (2014). Evidence that mifepristone, a progesterone receptor antagonist, can cross the blood brain barrier and provide palliative benefits for glioblastoma multiforme grade IV. *Anticancer Research*.

[26] Authier A., Farrand K. J., Broadley K. W. R. (2015). Enhanced immunosuppression by therapy-exposed glioblastoma multiforme tumor cells. *International Journal of Cancer*.

[27] Check J. H., Nazari P., Goldberg J., Yuen W., Angotti D. (2001). A model for potential tumor immunotherapy based on knowledge of immune mechanisms responsible for spontaneous abortion. *Medical Hypotheses*.

[28] Waziri A. (2010). Glioblastoma-derived mechanisms of systemic immunosuppression. *Neurosurgery Clinics of North America*.

[29] Raghupathy R., Al-Mutawa E., Al-Azemi M., Makhseed M., Azizieh F., Szekeres-Bartho J. (2009). Progesterone-induced blocking factor (PIBF) modulates cytokine production by lymphocytes from women with recurrent miscarriage or preterm delivery. *Journal of Reproductive Immunology*.

[30] Check J. H., Dix E., Sansoucie L. (2009). Support for the hypothesis that successful immunotherapy of various cancers can be achieved by inhibiting a progesterone associated immunomodulatory protein. *Medical Hypotheses*.

[31] Cheng L., Bao S., Rich J. N. (2010). Potential therapeutic implications of cancer stem cells in glioblastoma. *Biochemical Pharmacology*.

[32] Riemenschneider M. J., Reifenberger G. (2009). Astrocytic tumors. *Recent Results in Cancer Research*.

[33] Atif F., Yousuf S., Stein D. G. (2015). Anti-tumor effects of progesterone in human glioblastoma multiforme: role of PI3K/Akt/mTOR signaling. *Journal of Steroid Biochemistry and Molecular Biology*.

[34] Atif F., Patel N. R., Yousuf S., Stein D. G. (2015). The synergistic effect of combination progesterone and temozolomide on human glioblastoma cells. *PLoS ONE*.

[35] Cohen R. A., Check J. H., Dougherty M. P. (2016). Evidence that exposure to progesterone alone is a sufficient stimulus to cause a precipitous rise in the immunomodulatory protein the progesterone induced blocking factor (PIBF). *Journal of Assisted Reproduction and Genetics*.

[36] Bogdan A., Polgar B., Szekeres-Bartho J. (2014). Progesterone induced blocking factor isoforms in normal and failed murine pregnancies. *American Journal of Reproductive Immunology*.

[37] Srivastava M. D., Thomas A., Srivastava B. I. S., Check J. H. (2007). Expression and modulation of progesterone induced blocking factor (PIBF) and innate immune factors in human leukemia cell lines by progesterone and mifepristone. *Leukemia and Lymphoma*.

[38] Kozma N., Halasz M., Palkovics T., Szekeres-Bartho J. (2006). The progesterone-induced blocking factor modulates the balance of PKC and intracellular Ca^++^. *American Journal of Reproductive Immunology*.

[39] Senft C., Priester M., Polacin M. (2011). Inhibition of the JAK-2/STAT3 signaling pathway impedes the migratory and invasive potential of human glioblastoma cells. *Journal of Neuro-Oncology*.

[40] Priester M., Copanaki E., Vafaizadeh V. (2013). STAT3 silencing inhibits glioma single cell infiltration and tumor growth. *Neuro-Oncology*.

[41] Canellada A., Alvarez I., Berod L., Gentile T. (2008). Estrogen and progesterone regulate the IL-6 signal transduction pathway in antibody secreting cells. *Journal of Steroid Biochemistry and Molecular Biology*.

[42] Thorne A. H., Zanca C., Furnari F. (2016). Epidermal growth factor receptor targeting and challenges in glioblastoma. *Neuro-Oncology*.

